# An Assessment of the Relationship between Structural and Functional Imaging of Cerebrovascular Disease and Cognition-Related Fibers

**DOI:** 10.1155/2020/4347676

**Published:** 2020-01-20

**Authors:** Xiaoping Tang, Xinlan Xiao, Jianhua Yin, Ting Yang, Bingliang Zeng

**Affiliations:** ^1^Department of Radiology, Second Affiliated Hospital of Nanchang University, Nanchang 330006, Jiangxi, China; ^2^Department of Radiology, Jiangxi Provincial People's Hospital Affiliated to Nanchang University, Nanchang 330006, Jiangxi, China

## Abstract

In order to assess the relationship between structural and functional imaging of cerebrovascular disease and cognition-related fibers, this paper chooses a total of 120 patients who underwent cerebral small vessel disease (CSVD) treatment at a designated hospital by this study from June 2013 to June 2018 and divides them into 3 groups according to the random number table method: vascular dementia (VaD) group, vascular cognitive impairment no dementia (VCIND) group, and noncognition impairment (NCI) group with 40 cases of patients in each group. Cognitive function measurement and imaging examination were performed for these 3 groups of patients, and the observation indicators of cognitive state examination (CSE), mental assessment scale (MAS), clock drawing test (CDT), adult intelligence scale (AIS), frontal assessment battery (FAB), verbal fluency test (VFT), trail making test (TMT), cognitive index (CI), white matter lesions (WML), third ventricle width (TVW), and frontal horn index (FHI) were tested, respectively. The results shows that the average scores of CSE, MAS, AIS, and VFT in the VaD and VCIND group are lower than those of the NCI group and the differences are statistically significant (*P* < 0.05); the average scores of FAB, TMT, and CI in the VaD group are higher than those of the VCIND group and the differences are also statistically significant (*P* < 0.05); the average scores of FHI and TVW in the VaD group are lower than those of the VCIND and NCI group with statistically significant differences (*P* < 0.05); the average scores of WML, CDT, and AIS in the VaD group are higher than those of the VCIND and NCI group with statistically significant differences (*P* < 0.05). Therefore, it is believed that the structural and functional imaging features of cerebrovascular disease are closely related to cognition-related fibers, and the incidence of white matter lesions is closely related to the degree of lesions and cognitive dysfunction of cerebral small vessel disease, in which a major risk factor for cognitive dysfunction in patients with small blood vessels is the severity of white matter lesions; brain imaging and neuropsychiatric function assessment can better understand the relationship between cerebrovascular disease and cognitive impairment. The results of this study provide a reference for the further research studies on the relationship between structural and functional imaging of cerebrovascular disease and cognition-related fibers.

## 1. Introduction

Cerebral small vessel disease (CSVD) refers to a group of lesions in intracranial arterioles, arterioles, anterior capillaries, and venules due to different causes and their clinical manifestations mainly include focal cerebral infarction, various lacunar syndromes, and cognitive dysfunction with imaging findings of vascular dementia (VaD), lacunar infarction (LI), cerebral white matter lesion (WML), and cerebral microbleeds (CMB). The vascular dementia (VaD) is mainly caused by a series of brain vascular risk factors, which lead to dementia syndrome and clinical manifestations of cognitive dysfunction [1]. For subcortical ischemic VaD, it is a subtype of VaD, which is better in homogeneity, and is also a type of dementia in which cognitive function is impaired due to severe white matter lesions under the cortex [2]. In neuroimaging, white matter lesions around the lateral ventricle are typical, but the cortex is rarely involved. Subcortical ischemic vascular disease (SIVD) is caused by small blood vessel lesions leading to lacunar infarction and white matter lesions, and the pathogenesis of VaD remains to be further elucidated [3]. Therefore, some modern research techniques and methods, such as brain imaging and neuropsychiatric functional assessment, can better understand the relationship between cerebrovascular disease and cognitive impairment [4].

Although structural magnetic resonance imaging (MRI) has been a very useful tool to help physicians to accurately detect gross anatomical changes associated with a range of clinical disorders and typical developmental processes, while functional magnetic resonance imaging enables researchers to map different cognitive structures of specific brain regions in conscious and task-seeking individuals. Functional magnetic resonance is a relatively new technology, and it is clear that this technology may drive the rapid advancement of psychology and neuroscience [5]. Clinically, the ADC value of patients with cerebrovascular disease will increase to varying degrees when MRI is used; the ADC value mainly reflects the diffusion of water molecules in the direction of the gradient magnetic field, which cannot fully reflect specificity between different tissues. Functional magnetic resonance not only has excellent spatial resolution, but it also provides researchers with a window that allows researchers to observe brain changes in subjects performing cognitive tasks. Although the voxel-based morphology (VBM) analysis method can be used not only to analyze the difference in the gray matter structure between Alzheimer's disease (AD) patients and normal individuals but also to suggest changes in white matter structure, but whether white matter atrophy exists and its degree of damage cannot pass [6]. This method is completely deterministic, suggesting that macroscopic white matter damage detected by voxel-based morphology (VBM) does not fully reflect microscopic white matter integrity changes [7]. Diffusion tensor imaging (DTI) is a more sensitive imaging method for detecting white matter microscopic tissue damage, and it has been confirmed that DTI parameters are more closely related to MRI image volume and cognition [8].

In order to assess the relationship between structural and functional imaging of cerebrovascular disease and cognition-related fibers, this paper chooses a total of 120 patients who underwent cerebral small vessel disease treatment at a designated hospital by this study from June 2013 to June 2018 and divides them into 3 groups according to the random number table method: vascular dementia group, vascular cognitive impairment no dementia group, and noncognition impairment group with 40 cases of patients in each group. Cognitive function measurement and imaging examination were performed for these 3 groups of patients, and the observation indicators of cognitive state examination, mental assessment scale, clock drawing test, adult intelligence scale, frontal assessment battery, verbal fluency test, trail making test, cognitive index, white matter lesions, third ventricle width, and frontal horn index were tested, respectively. The detailed chapters are organized as follows: Section 2 presents research materials and methods; Section 3 performs results analysis; Section 4 assesses the relationship between structural and functional imaging of cerebrovascular disease and cognition-related fibers; Section 5 is discussion; and Section 6 is conclusion.

## 2. Materials and Methods

### 2.1. General Materials

A total of 120 patients who underwent SIVD treatment at a designated hospital by this study from June 2013 to June 2018 were selected as study subjects and were divided into 3 groups according to the random number table method: vascular dementia (VaD) group, vascular cognitive impairment no dementia (VCIND) group, and noncognition impairment (NCI) group with 40 cases of patients in each group. The VaD group contains 23 males and 17 females; their ages are in 55–83 years old with an average age of (66.27 ± 5.67) years old; their daily living ability scale (ADL) scores are in 18–45 with an average score of (28.17 ± 1.59); their cognitive state examination (CSE) scores are in 10–23 with an average score of (20.06 ± 3.84); their mental assessment scale (MAS) scores are in 11–26 with an average score of (24.52 ± 2.71); their educational experiences include 2 cases of primary school and below, 22 cases of junior high school education, and 16 cases of high school education and above (see Table 1). The VCIND group contains 24 males and 16 females; their age are 52–85 years old with an average age of (66.32 ± 5.64) years old; their ADL scores are 16–44 with an average score of (28.04 ± 1.62); their CSE scores are 11–23 with an average score of (19.79 ± 3.71); their MAS scores are 10–25 with an average score of (24.57 ± 2.63); their educational experiences include 5 cases of primary school and below, 17 cases of junior high school education, and 28 cases of high school education and above (see Table 1). The NCI group contains 20 males and 20 females; their ages are 53–86 years old with an average age of (66.48 ± 5.67) years old; their ADL scores are 18–44 with an average score of (27.83 ± 1.67); their CSE scores are 12–25 with an average score of (20.14 ± 3.49); their MAS scores are 11–25 with an average score of (24.33 ± 2.57); their educational experiences include 7 cases of primary school and below, 15 cases of junior high school education, and 28 cases of high school and above (see Table 1). There are no significant differences in the general data of the three groups (*P* < 0.05), and the data are comparable. This study was reviewed and approved by the hospital medical ethics committee.

### 2.2. Inclusion and Exclusion Criteria

Inclusion criteria: (1) patients who meet SIVD MRI diagnostic criteria and SIVD diagnostic criteria developed in the expert consensus on vascular cognitive impairment; (2) patients with acute ischemic cerebrovascular disease more than 1 time and more than 3 months; (3) patients whose brain MRI diagnosis shows white matter damage and lacunar infarction prominent; (4) patients with cognitive impairment; (5) patients who were passed by the hospital ethics committee and themselves or their family members were informed and voluntarily signed informed consent.

Exclusion criteria: (1) patients with severe visual and hearing impairment, severe physical weakness, or aphasia affecting the examination or failing to cooperate; (2) patients with mental illness or disturbance of consciousness; (3) patients who had already presented cognitive dysfunction and their assessment scores exceeding 2 points or a short caregiver questionnaire totalling more than 56 points; (4) patients who have pseudocognitive impairment or dementia caused by depression, anxiety, or AD; (5) patients whose cognitive dysfunction caused by other diseases, including infection, cancer, poisoning, metabolic diseases, and congenital mental retardation; (6) patients with severe heart, liver, lung, kidney and other organ failure, and therefore the examination cannot be completed; (7) patients who have MRI scan contraindications or are unable to scan; (8) patients whose imaging examination indicates the presence of other intracranial diseases, including cerebral hemorrhage and subarachnoid hemorrhage; (9) patients or their family are not willing to participate in the experiment.

### 2.3. Cognitive Function Determination Methods

All patients were enrolled with basic information such as name, age, gender, and education level. The cognitive function rating scale is mainly divided into four parts: (1) simple mental state examination CSE; the table consists of 40 questions, a total of 40 items; each answer is correct 1 point, the answer is wrong or the answer does not know the score 0; the total score of the scale is 0–30 points; (2) the meaning of the word is composed of 40 questions, each score is 1 or 0 points, out of 40 points (3) digital recitation is a subtest consisting of two parts: the back and back of the number, each score is 2, 1, and 0, and the total score is the sum of the two, with a score of 40; (4) line connection test is divided into two parts: A and B; A is 40 numbers in sequence and B puts 40 numbers in square and round and requires two patterns to be alternately and sequentially connected and the time spent in two parts are calculated. Pearson correlation analysis software was used to analyze the relationship between imaging findings and cognitive dysfunction of cerebral small vessel disease.

### 2.4. Imaging Examination Methods

All patients were examined by magnetic resonance imaging to obtain diffusion coefficient (ADC) parameters; after admission, the MRI was performed with a Philips 3.0T magnetic resonance instrument. The relevant parameters were set according to the actual situation of each patient; T1-weighed image (T1W1): time of repetition (TR) is 1800 ms, echo time (TE) is 30 ms; T2-weighed image (T2W1): TR is 2000 ms, TE is 100 ms; fluid attenuated inversion recovery (FLAIR): TR is 10000 ms, TE is 125 ms, TE is 100 ms; matrix 120 × 124, layer thickness is 4 mm, and pitch is 0.3 mm with continuous 80 layer scans. Image processing: the obtained route is processed by Philips proprietary software, and then the image is subjected to the dispersion alignment software to obtain ADC parameters.

### 2.5. Observation Indicators

Observation indicators include cognitive state examination (CSE), mental assessment scale (MAS), clock drawing test (CDT), adult intelligence scale (AIS), frontal assessment battery (FAB), verbal fluency test (VFT), color word test (CWT), trail making test (TMT), cognitive index (CI), white matter lesions (WML), third ventricle width (TVW), and frontal horn index (FHI).

### 2.6. Indicator Examination Methods

**Figure 1:**
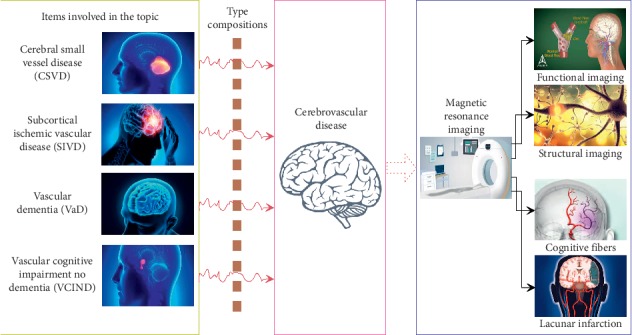
Framework of detecting the relationship between structural and functional imaging of cerebrovascular disease and cognition-related fibers.

The main indicator examination workflow is shown in Figure 1 and is described as follows:

  TMT: the project is a subtest of the neuropsychological test; part A connects 40 Arabic numerals in sequence, and part B alternates numbers and letters; the test only performs part A, recording the completion time.
  FAB: it is a trial of the frontal dysfunction including similarity, lexical fluency, motion sequence testing, inconsistency instructions, inhibitory control and gripping behaviour and six subtests; the score for each item is between 0 and 4 points, with a maximum total score of 20 points; a higher score indicates better performance.
  CDT: on a blank sheet of paper, the subject draws a dial and is asked to write all the numbers; the tester indicates the pointer at 11 : 10; the tester evaluates the subject, including whether the contour of the watch is complete and whether the pointer is correct and the total score is 3 points, and the correction and rubbing are not deducted.
  CWT: a set of three test cards and the test contents of the three cards are arranged in a 8 × 8 matrix, and the time (s) used to read the three cards is recorded separately; the A card consists of red dots, yellow dots, blue dots, and green dots, and reads the dot color; the B card is composed of four characters and the characters are red; the four colours of yellow, blue and green are printed in monochrome, and the color of characters is required to be read; the C card is four characters of red, yellow, blue, and green and the printing colours are these four colours, and the printing colours are different from the meaning of the characters.

### 2.7. Statistical Methods

Data analysis was performed using SPSS 19.0 statistical software. The data of the measurement data were expressed as mean ± standard deviation ($\bar{x}$ ± *s*) and the *t* test was used to compare the two groups. The statistical analysis was performed after the normal distribution was not consistent with the normal distribution and one-way analysis of variance was used for comparison between groups; the LSD-*t* test was used for comparison between groups. *P* < 0.05 was considered statistically significant.

## 3. Result Analysis

### 3.1. Comparison of Overall Cognitive Function in Three Groups of Patients

At present, there is a view that the number of lesions in the lacunar infarct is more significant than the number and volume of lesions. This study also found a correlation between cognitive function and lacunar infarction and there was a statistically significant difference in the number of subcortical white matter lacunar infarction between the three groups which may be due to lacunar infarction in the white matter (see Table 2). The lesion cuts off the fibrous relationship between certain structures under the cortex and the prefrontal cortex, the frontal cortex, and the cingulate gyrus, thereby inhibiting some of the functions associated with cognition in the frontal cortex and further leading to dementia. Patients with congestive heart failure are at high risk for cognitive dysfunction, suggesting that a decrease in cardiac output may affect the blood and nutritional supply of the brain and patients with low left ventricular dysfunction have a marked decline in cognitive function. This study did not find that cognitive impairment was associated with the number of lacunar infarct lesions in the putamen, caudate nucleus, and globus pallidus; while the number of lacunar infarct lesions in the thalamus between the three groups was different, but no statistically significant speculation may be related to the small number of cases in this study. In this study, compared with the noncognitive disorder group, the WML scores of the mild cognitive impairment group and the vascular dementia group generally showed an increasing trend, suggesting that there is a correlation between the degree of cognitive impairment and the degree of total WML lesions.

There are many terms describing white matter high signal, such as leukoaraiosis, white matter lesions, white matter high signal, and leukoencephalopathy. The MRI of leukoaraiosis is defined as high signal in sequence, often difficult to find in sequence or mildly low signal, mainly distributed around the ventricles, bilateral white hemisphere deep white matter, basal ganglia, pons, and occasionally in the cerebellum white matter areas such as brainstem. The leukoaraiosis is closely related to the occurrence of dementia and has been found that leukoaraiosis can accelerate the occurrence of dementia through ischemic injury of the cortex and directly aggravate the pathological changes of AD. It is hypothesized that the angiogenic white matter high signal excludes white matter lesions caused by other nonangiogenic diseases, such as multiple sclerosis and white matter malnutrition [5]. The imaging feature is a high signal with a point-like, patchy, or convergent weighting and the weighted signal is equal or low, and the signal depends on the image sequence parameters and the degree of lesion. Some scholars have pointed out that hypothetical angiogenic white matter high signal has a high heritability, making it an intermediate marker for clinical research and screening for new risk factors for stroke or dementia. Clinical case resources and rapid development of imaging techniques study its pathogenesis and combine with clinical risk factors.

Brain atrophy can manifest as symmetric or asymmetrical, extensive or localized atrophy, and may be tissue selective. The imaging manifestation is that the brain volume becomes smaller, and it has nothing to do with the local trauma and infarction of the brain visible to the naked eye. The pathological changes of brain atrophy are heterogeneous, which represents neuronal loss, cortical thinning, reduced white matter sparseness, arteriosclerosis, and secondary neuronal degeneration [9]. The brain atrophy may be associated with cognitive impairment, and studies have found that the volume of nucleus accumbens, amygdala, caudate nucleus, thalamus, and brainstem is closely related to cognition. Several patients with transient ischemic attack or suspected stroke participated in the survey and several patients had atrophy of medial temporal lobe, and the study found that medial temporal lobe atrophy was associated with memory, naming, sensory ability, executive ability and speed, and attention. Cerebral microinfarction refers to small infarction caused by ischemia, and high-resolution magnetic resonance can detect large microinfarction. Based on population and autopsy studies, brain microinfarction is associated with cognitive decline and the mechanism of cognitive influence is not clear, which may be related to the destruction of the brain contact fiber network and brain microinfarction is an important bridge linking cerebrovascular disease and dementia.

### 3.2. Comparison of Imaging Findings of Three Groups of Patients

Previous studies have indicated that patients with a more uniform reduction in cortical density include the primary motor cortex, frontal lobe, temporal lobe, marginal lobe, and lateral central lobules. The results of this study suggest that compared with the healthy control group, the gray density of the bilateral central anterior gyrus is significantly lower than that of the normal control group and the gray matter density of other brain regions is not significantly abnormal (see Table 3). The bilateral central anterior gyrus is the motor area of the cerebral cortex and this part of the cortical dysfunction can lead to dyskinesia in patients; this result supports the core pathological changes of amyotrophic lateral sclerosis. Lacunar infarction, white matter lesions, and basal ganglia lesions are closely related to stroke and transient ischemic attacks; they are closely related to the presence of gait disorders in patients. The study sample size is small, the patients included in the early stage of the disease, the magnetic resonance scanning parameters used in previous studies, and the limitations of the magnetic resonance processing technology itself may cause the analysis results of this study to be inconsistent with previous reports. The enrolment of patients in the early stages of the disease is the main reason for the large difference between the results of this study and the past. However, cross-sectional studies grouped by disease severity or subsequent longitudinal follow-up studies of this subject are needed to confirm this reasoning. The thickness of the cortex can reflect the neuropathological characteristics of the disease more sensitively than the gray matter density or volume which is an imaging index that studies the morphological changes of the cerebral cortex with high reliability.

Conventional MRI imaging showed that mild to moderate WML had no significant effect on cognitive function. The cognitive function of mild to moderate WML depends mainly on the total number of basal and thalamic regions LI; the basal ganglia and thalamus are key sites in the development of LI, which can better predict cognitive decline. LI distributed in the white matter of the brain extensively interferes with the structure of the white matter, and chronic inflammatory processes, loss of myelin, and axonal degeneration may cause more severe cognitive decline due to such interference. The pathogenesis of WML progresses from the subcortical white matter to the white matter adjacent to the ventricles and cystic LI may be involved in extensive WML under the cortex, and the number of LI in subcortical WML cannot be fully recognized. Analysis of the MRI results of LI and WML in the basal and thalamic regions showed that the severity of WML and the number of basal and thalamic regions LI were related to the extent of cognitive impairment. This study found that severe WML or basal ganglia and multiple lymphoid in the thalamus area can cause cognitive decline. Probably because cognitive function depends on neural network function, both WML and LI have a phenomenon of neural network interruption, the so-called separation syndrome, which has a decline in cognitive function [10].

Perivascular spaces (PVS) refer to the filling of fluid around the cerebral perforating arterioles and venules, and its diameter is usually less than 3 mm. The imaging findings are T1W1 low signal, T2W1 high signal, and FLAIR low signal. It plays an important role in the formation of a discharge channel for metabolic waste and liquid in the brain. Whether or not PVS have clinical significance is controversial and some studies suggest that these gaps do not represent damage, and some studies have shown that PVS is associated with decreased cognitive function. Previous studies found that there was no independent association between PVS and cognitive impairment in people with ischemic stroke and transient ischemic attack large PVS is associated with subcortical infarction, increased volume, and microbleeds. It indicates that there may be a correlation between PVS and AD, and the study found that PVS in the basal ganglia were associated with high levels of cerebrospinal fluid; PVS in the semioval centre and PVS in the basal ganglia were different, but were associated with cerebral amyloid angiopathy and hypertensive vasculopathy. The former is more associated with AD than the PVS in the white matter region and the basal ganglia PVS.

## 4. Relationship between Cerebrovascular Disease Imaging and Cognition-Related Fibers

### 4.1. Relationship between Structural Imaging of Cerebrovascular Disease and Cognition-Related Fibers

**Figure 2:**
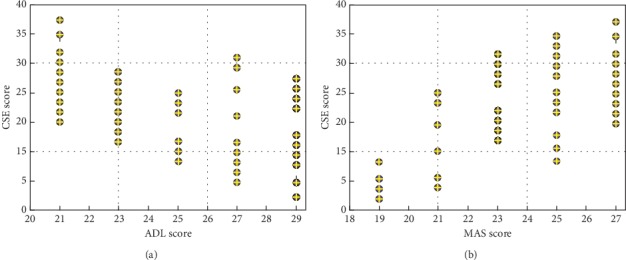
Analysis results of the relationship between structural imaging of cerebrovascular disease and cognition-related fibers. (a) The relationship between CSE and ADL score in the VaD group of patients. (b) The relationship between CSE and MAS score in the VaD group of patients.

The results suggest that the medial temporal lobe memory system, especially the integrity of hippocampus and cortical-associated fibers, is closely related to cognitive function. The medial temporal lobe memory system, also known as the hippocampus memory system or the marginal memory system, is located in the ventral aspect of the temporal lobe and is composed of the hippocampus formation and adjacent cortex. Nearly two-third of the afferent fibers of the hippocampus are from the parasitic cortex and the hippocampus cortex (see Figure 2(a)). The parasitic cortex and the hippocampus cortex have extensive bidirectional fiber connections with the frontal, temporal, and parietal cortex. The information in the hippocampus is the main pathway, and the entorhinal cortex is a temporary integration zone in the cortical liaison zone. In the course of AD, severe pathological changes block the transmission of information in different brain regions, the damage to the circuit is different in different cortex, and the two major cortical systems that play an important role in cognitive memory are particularly selective [11]. One is the fiber connection between the hippocampus and the adjacent cortical structure in the temporal lobe, which is reflected in the atrophy of the parasitic cortex and the expansion of the hook spacing representing the hippocampus formation and atrophy of the hippocampus cortex (see Figure 2(b)). The second is the basal forebrain energy system. These circuits show severe degenerative changes in the early stages of AD, which may be the cause of significant memory loss in the early stages of AD.

The integrity of cerebral blood circulation is closely related to the occurrence and prognosis of cerebral infarction, especially in the event of internal carotid artery stenosis or occlusion. In patients with severe stenosis or occlusion of the unilateral internal carotid artery, anterior communicating artery opening is a predictor of hemodynamic preservation, whereas no arterial flow is a sign of hemodynamic damage after collateral circulation or only blood flow. Secondary collateral circulation occurs when the primary collateral circulation is not developed or is still unable to maintain normal perfusion, and is also important, including pia joint anastomosis and ophthalmic artery. As the pia matter undulates on the surface of the sulci, it belongs to the secondary collateral circulation, and the diameter and number of anastomotic vessels are extremely large variability in which blood flow can be bidirectional with hemodynamic changes and metabolic needs in the anastomotic region. Clinically, the infarction, stenosis or occlusion caused by secondary branch lesions occurs in the anterior and posterior cerebral arteries, and the circulation is difficult to compensate. The opening of the pial-macular anastomosis will play a major role, and the opening of the pia matter anastomosis is a reliable predictor of the good outcome of ischemic cerebrovascular disease.

As a common type of cerebral infarction, LI is mainly caused by hypertension, diabetes, and other factors leading to changes in the structure of small arteries in the brain, and the cerebral blood flow automatic regulation mechanism is impaired, eventually leading to cerebral infarction. It is characterized by small infarct size and multiple occurrences, and obvious cognitive dysfunction can occur without obvious signs of the nervous system, for example, the lesion in the posterior horn of the sac is associated with impaired executive function. The lesions in the frontal lobe are associated with memory impairment and executive function, coding, and management information dysfunction; occipital cavity and visual occlusion lesions are related; temporal lobe lesions are associated with memory, language, hearing, and affective disorders; lesions in the anterior thalamus are associated with amnesia. Studies have shown that the number, volume, and location of LI are independent predictors of treatment speed and performance impairment in symptomatic stroke. This study found that the CSE score and MAS score in the simple LI group were lower than those in the control group, indicating that the occurrence of LI does have a certain effect on cognitive dysfunction in the elderly and the plasma cognitive fiber level in the vascular dementia group was significantly higher; the increase in cognitive fiber was significantly related to the patient's oral memory disorder. The possible pathogenesis is LI destruction and information processing, speed, memory, and executive function related to the frontal-cortical loop [12].

### 4.2. Relationship between Functional Imaging of Cerebrovascular Disease and Cognition-Related Fibers

**Figure 3:**
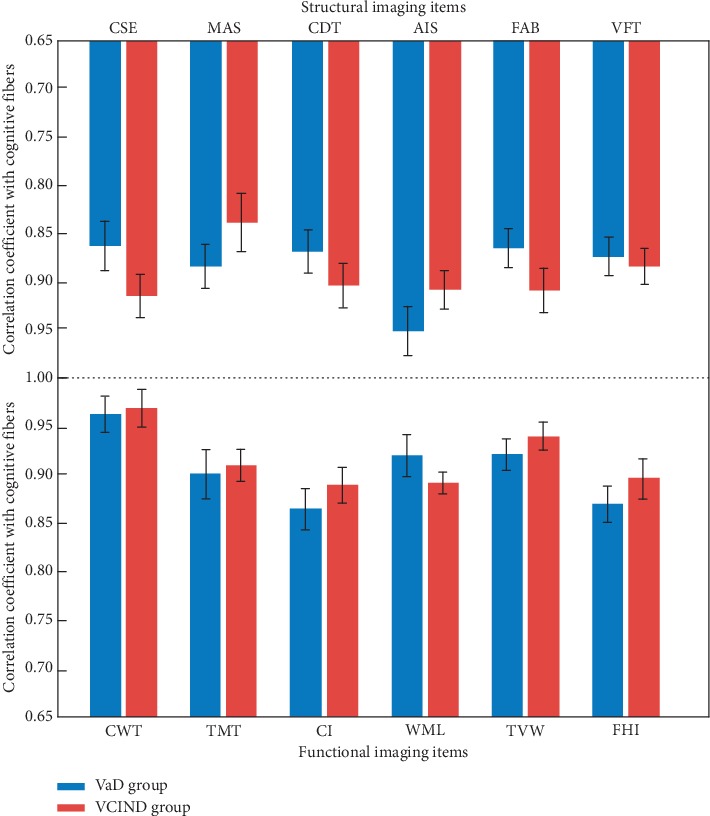
Result analysis of the relationship between functional imaging of cerebrovascular disease and cognition-related fibers (for the abbreviations, refer to the context).

Patients with cerebral small vessel disease are often accompanied by imaging changes and strengthening MRI can predict the degree of cerebral small vessel injury and guide clinical treatment. MR diffusion imaging is a commonly used imaging method in patients with cerebral small vessel disease; this method uses the flow of water molecules in the body of MRI to map the internal structure of the brain, which not only clearly shows the small changes in the brain, but also more accurate. It describes the changes in the blood flow of intracranial cerebrospinal fluid and small blood vessels while conventional magnetic resonance cannot reflect whether the diseased axon is damaged or not, and the MR can relatively determine whether the white matter fiber bundle is damaged or not and can assess the severity of the disease. Studies have shown that patients with cerebral small vessel disease can cause neuronal axon loss, resulting in the proliferation of glial cells and clinically, and have different degrees of ADC values when using MRI. The reason for this phenomenon is multifaceted because the ADC value mainly reflects the dispersion of water molecules in the direction of the gradient magnetic field; it cannot completely react to the specificity between different tissues (see Figure 3).

In the central nervous system, the diffusion of water molecules is affected by cell structures such as cell membranes, axonal membranes, and cytoskeleton and is also affected by natural barriers such as myelin, white matter fiber bundles, and protein macromolecules. There is a significant difference in the dispersion of the various parts of the central nervous system, and this difference is also the basis of diffusion tensor imaging. It is precisely because the dispersion of water molecules is a spontaneous physical phenomenon, so the scanning of the diffusion tensor does not require a specific cognitive task, and the data obtained by the subjects with or without stimulation is relatively stable. Diffusion tensor is currently the only noninvasive method for effectively observing and tracking white matter fiber bundles, which can show the anatomical and pathological processes of white matter fibers and indirectly evaluate the integrity of white matter fibers [13]. Compared with the traditional sequence of magnetic resonance imaging, the diffusion tensor has many advantages, such as scanning time period, more information obtained, better image quality, sensitive to changes in microstructure and function, and no need to use radioactivity, tracer, and high security.

Cognitive fibers and vascular lesions caused by atherosclerosis are not difficult to identify, and atherosclerosis is mainly seen in the elderly. The lesions are mainly located in the proximal end of the artery or in the bifurcation of the arteries, and there are many risk factors for cerebrovascular disease such as diabetes, hypertension, and hyperlipidemia found in young women; the lesions involve the middle and distal end of the artery, and there are many risk factors for cerebrovascular disease. The clinical symptoms of the patient are related to the degree of stenosis of the affected artery and also to the location of cognitive fiber lesions, involving the carotid or vertebral artery cognitive fibers, can be expressed as black, hemiplegia, and cranial neuropathy without any clinical symptoms. The affected blood vessels expand in a saclike manner, and the blood flow is slow or eddies in the local flow. Platelets, red blood cells, etc. form aggregates to form thrombus, and emboli fall off to cause cerebral embolism; severe stenosis or occlusion of the affected blood vessels can cause the blood flow in the distal cerebral tissue insufficient perfusion, causing hypoperfusion cerebral infarction. The lesions can involve aneurysms caused by intracranial blood vessels, subarachnoid hemorrhage caused by aneurysm rupture, and it causing stroke episodes combined with intracranial vascular dissection [14].

## 5. Discussion

### 5.1. Correlation between the Scores of Neuropsychological Scales and the Degree of White Matter Lesions

The influence of the location of lacunar infarction on cognitive function has been widely concerned; infarction in some key areas is more likely to lead to severe cognitive dysfunction which is recognized by most researchers. Case reports of a single cavity lesion in the strategic site have increased significantly, and cognitive impairment has occurred in the acute phase of stroke.  Table 4 shows the correlation analysis between the neuropsychological scale score and degree of white matter lesions. Brain activity as a whole, imaging features are high signals that are spotted, patchy, or fused on a weighted basis, and different behaviours are associated with different brain functional parts. For example, when the executive function is compromised, it is often suggested that the site of destruction is located in the subcortical loop associated with the frontal lobe [15]. The loop inside the dorsal prefrontal lobes is most important for performing functions and the medial cortex of the prefrontal cortex serves as the starting point of this loop. The nerve fibers that are emitted are first connected to the dorsal medial side of the caudate nucleus and then projected to the old striatum, reaching the nucleus of the thalamus, and finally returning, the cerebral cortex to the dorsal medial side of the prefrontal cortex. However, in this loop, small penetrating arteries supply blood to the white matter and gray matter structure, so ischemic diseases are easily produced in this loop.

**Figure 4:**
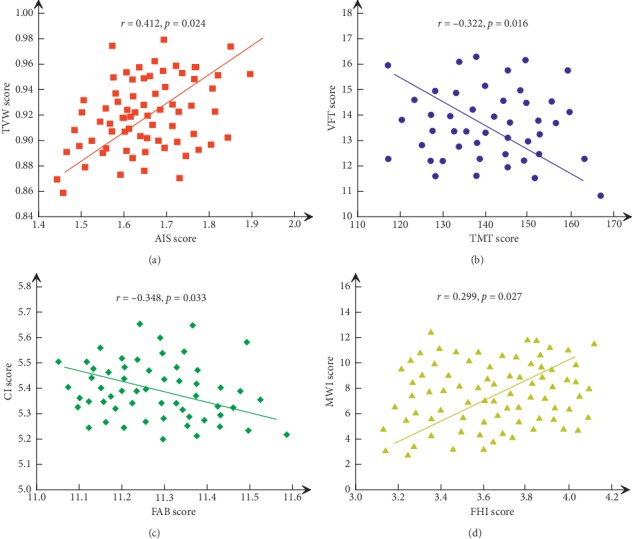
Correlation between the scores of neuropsychological scales and the degree of white matter lesions. (a) The relationship between TVM and AIS score. (b) The relationship between VFT and TMT score. (c) The relationship between CI and FAB score. (d) The relationship between MWL and FHI score. “r” and “p” were correlation coefficient and degree of fit, respectively.

Related studies have found that cardiovascular events, carotid plaque formation, and peripheral vascular arteriosclerosis are independent risk factors for cognitive impairment. The study found that ischemic heart disease is an important factor in the aggravation and death of AD, and long-term chronic arrhythmia and cardiac insufficiency can cause dementia caused by ischemia and hypoxia. Some studies have shown that patients with congestive heart failure have a high risk of cognitive dysfunction, suggesting that decreased cardiac output may affect the blood and nutrient supply of the brain; another study suggests that cognitive function is significantly reduced in patients with low left ventricular function (see Figure 4). There is no clear conclusion about the direct relationship between coronary heart disease and cognitive function and the effect of stroke on cognitive function is obvious. The decline in cognitive function caused by brain tissue loss due to the formation of intracerebral arterial infarction is called multi-infarct dementia or vascular dementia. Previous studies on vascular dementia have focused on cerebrovascular disease, and studies on cognitive impairment caused by other vascular lesions are relatively lacking. Because the detection of vascular lesions in the brain is relatively difficult and changes in coronary heart disease or peripheral arterial occlusive disease are easily identified, it can be used as a risk factor for cerebrovascular disease.

Secondary collateral circulation occurs when the primary collateral circulation is poorly compensated in patients with severe internal carotid artery stenosis or occlusion, and secondary collateral circulation and open pial obstruction is an important collateral compensatory pathway. The presence of secondary collateral circulation did not reduce the incidence of infarct events, but cognitive impairment of the internal carotid artery stenosis or occlusion in the presence of infarction was more pronounced than in patients without infarction. The study analyzed the risk factors of severe internal stenosis or occlusion of the internal carotid artery and used neuroimaging to observe the damage of brain tissue structure, collateral circulation, and cerebral blood flow changes and evaluate cognitive function to explore the severe internal stenosis or occlusion of the internal carotid artery. The mechanism of circulatory compensation, cerebral blood perfusion, and cognitive dysfunction provides a basis for establishing an early clinical neuroimaging diagnostic evaluation system, suggesting that clinicians are not only in the early stage of patients with severe internal stenosis or occlusion of the internal carotid artery. The evaluation of the brain tissue structure should pay more attention to collateral circulation compensation, cerebral blood perfusion, and cognitive function changes, so as to formulate standardized individual treatment plans, and should conduct early screening and reasonable vascular cognitive dysfunction and intervention to improve the quality of life of patients [16].

### 5.2. Association Mechanism of Cerebrovascular Disease Imaging and Cognition-Related Fibers

**Figure 5:**
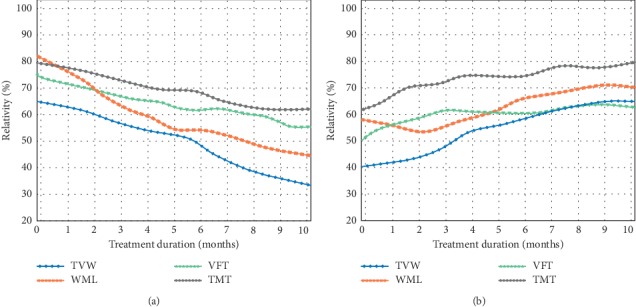
Evolution of key indicator relativity with treatment duration. (a) VaD group. (b) VCIND group.

Cerebral small vessel disease is a common type of disease in vascular cognitive impairment, and clinical studies have consistently shown that frontal white matter lesions and lacunar infarction are leading to further aggravation of patients, progressive cognitive impairment, and the gait is an important factor in abnormality, and the occipital leukoencephalopathy and lacunar infarction are very likely to cause symptoms such as convulsions and incontinence in patients with cerebral small vessel disease (see Figure 5). Related studies have shown that lacunar infarction, white matter lesions, and basal ganglia lesions are closely related to stroke and transient ischemic attack, and they are closely related to patients having gait disorders [17]. Cerebral small vessel disease is a disease that is seriously harmful to the brain function and systemic health of patients and is the main cause of stroke and cognitive decline in patients. White matter and lacunar infarction are the most important imaging manifestations of the disease, which is beneficial to the clinical combination of characteristics to understand the disease and the brain damage of patients, and then select the corresponding drugs for patients and improve the functional damage.

A meta-analysis showed a positive correlation between cognitive fiber levels and the incidence of vascular dementia; high cognitive fiber levels promote cognitive dysfunction in patients with existing white matter lesions. Compared with the AD patients, plasma cognitive fiber levels were significantly increased in the vascular dementia group; cognitive fiber elevation was significantly associated with oral memory impairment in patients. The patients with mild cognitive impairment with high cognitive fiber levels are more likely to develop dementia, suggesting that controlling plasma cognitive fiber levels in patients with mild cognitive impairment may help delay cognitive decline. The specific mechanisms by which cognitive fibers cause cognitive dysfunction are not yet clear. In the animal model of AD, it is found that the regional cognitive fiber level of axonal dystrophy is elevated, and the cognitive fiber level is moderately reduced, which can reduce neuronal damage and amyloid deposition in the brain. Elevated cognitive fibers can also promote changes in blood coagulation function, which in turn affect platelet aggregation and vascular endothelial cell function, increase leukoaraiosis, and cause cognitive decline. In addition, cognitive fibers can promote platelet aggregation, increase blood viscosity, and promote thrombosis. Increased cognitive fibers can aggravate the degree of intracranial and extracranial stenosis and increase the recurrence rate of cerebral infarction, thus increasing cognitive dysfunction [18].

White matter lesions have long been a widespread concern as a common brain imaging change and they usually coexist with stroke and neurodegenerative diseases. A large number of imaging studies have confirmed that most patients with AD have varying degrees of white matter lesions, and more importantly, more and more studies in recent years have shown that white matter lesions can be used as mild cognitive impairment or a neuroimaging marker of AD. However, severe white matter lesions are almost impossible to reverse or repair, and early recognition of mild white matter lesions is particularly important for the brain. As the pia matter undulates and spreads on the surface of the gyrus, it belongs to the secondary collateral circulation; the effects of early white matter lesions on cognition, brain structure and function, and their relationship in order to provide a basis for clinicians to better understand their pathogenic mechanisms [19]. In the past, some studies have pointed out that no significant cognitive impairment was found in mild white matter lesions and one of the reasons may be that multiple factors that are beneficial and unfavourable for maintaining normal cognitive function have multiple effects on the decline of neuroprotective mechanisms. Therefore, different degrees of cognitive decline occur in different stages of white matter lesions from mild to severe.

## 6. Conclusions

In order to assess the relationship between structural and functional imaging of cerebrovascular disease and cognition-related fibers, this paper chooses a total of 120 patients who underwent cerebral small vessel disease treatment at a designated hospital by this study from June 2013 to June 2018 and divides them into 3 groups according to the random number table method: vascular dementia group, vascular cognitive impairment no dementia group, and noncognition impairment group with 40 cases of patients in each group. Cognitive function measurement and imaging examination were performed for these 3 groups of patients, and the observation indicators of CSE, MAS, CDT, AIS, FAB, VFT, TMT, CI, WML, TVW, and FHI were tested, perceptively. All patients were examined by magnetic resonance imaging to obtain diffusion coefficient parameters; after admission, the MRI was performed with magnetic resonance spectroscopy. The relevant parameters were set according to the actual situation of each patient and the statistics were used and the data were analyzed by software SPSS 19.0; the data of the measurement data were expressed by the mean ± standard deviation and the *t* test was used for comparison between the two groups. The statistical analysis was performed after the normal distribution was not converted into a normal distribution, and the one-way analysis was used for comparison between groups. The LSD-*t* test was used to compare the two groups and the difference was statistically significant at *P* < 0.05.

The results shows that the average scores of CSE, MAS, AIS, and VTF in the VaD and VCIND group are lower than those of the NCI group and the differences are statistically significant (*P* < 0.05); the average scores of FAB, TMT, and CI in the VaD group are higher than those of the VCIND group and the differences are also statistically significant (*P* < 0.05); the average scores of FHI and TVW in the VaD group are lower than those of the VCIND and NCI group with statistically significant differences (*P* < 0.05); the average scores of WML, CDT, and AIS in the VaD group are higher than those of the VCIND and NCI group with statistically significant differences (*P* < 0.05). Therefore, it is believed that the structural and functional imaging features of cerebrovascular disease are closely related to cognition-related fibers, and the incidence of white matter lesions is closely related to the degree of lesions and cognitive dysfunction of cerebral small vessel disease, in which a major risk factor for cognitive dysfunction in patients with small blood vessels is the severity of white matter lesions. Imaging features are high signals that are spotted, patchy, or fused on a weighted basis, equal or low signals on a weighted basis, and their signals depend on the image sequence parameters and the degree of lesions. The integrity of cerebral blood circulation is closely related to the occurrence and prognosis of cerebral infarction, especially in the internal carotid artery stenosis or occlusion; in patients with severe stenosis or occlusion of the unilateral internal carotid artery, anterior communicating artery opening is a predictor of hemodynamics. The results of this study provide a reference for further research on the relationship between structural and functional imaging and cognition-related fiber relationship in cerebrovascular disease.

## Figures and Tables

**Table 1:** General information of SIVD patients in VaD, VCIND, and NCI group ($\bar{x}$ ± *s*).

Group	Average age (years old)	Gender (male/female)	ADL	CSE	MAS	Education level		
						PSB	JHS	HSA
VaD	66.27 ± 5.67	23/17	28.17 ± 1.59	20.06 ± 3.84	24.52 ± 3.84	2	22	16
VCIND	66.32 ± 5.64	24/16	28.04 ± 1.62	19.79 ± 3.71	24.57 ± 2.63	5	17	28
NCI	66.48 ± 5.67	20/20	24.33 ± 2.57	84.97 ± 5.31	24.33 ± 2.57	7	15	28
*P*	>0.05	>0.05	>0.05	>0.05	>0.05	>0.05		

PSB: primary school and below; JHS: junior high school; HAS: high school and above.

**Table 2:** Comparison of TVW, AIS, VFT, and TMT in the VaD, VCIND, and NCI patients ($\bar{x}$ ± *s*).

Group	Treatment time	TVW	AIS	VFT	TMT
VaD (*n* = 40)	Before treatment	0.96 ± 0.23	1.52 ± 0.49	10.27 ± 3.17	123.63 ± 57.73
	After 6 months of treatment	0.93 ± 0.21	1.36 ± 0.38	12.46 ± 2.19	122.15 ± 45.38
	After 12 months of treatment	0.84 ± 0.19	1.63 ± 0.11	15.13 ± 2.14	120.77 ± 54.69
VCIND (*n* = 40)	Before treatment	0.91 ± 0.22	1.68 ± 0.39	11.14 ± 3.12	123.55 ± 56.87
	After 6 months of treatment	0.88 ± 0.21	1.57 ± 0.27	13.03 ± 2.06	121.89 ± 45.44
	After 12 months of treatment	0.82 ± 0.18	1.21 ± 0.63	16.44 ± 2.16	120.32 ± 54.76
NCI (*n* = 40)	Before treatment	0.80 ± 0.19	1.79 ± 0.41	15.03 ± 3.06	123.46 ± 46.35
	After 6 months of treatment	0.81 ± 0.17	1.80 ± 0.24	15.75 ± 2.04	115.57 ± 55.01
	After 12 months of treatment	0.79 ± 0.16	1.87 ± 0.36	16.72 ± 2.13	111.47 ± 45.32
*F*/*P* VaD group value		0.163/0.028	0.325/0.003	0.754/0.003	0.855/0.016
*F*/*P* VCIND group value		0.174/0.005	0.542/0.018	0.584/0.027	0.585/0.027
*F*/*P* NCI group value		0.164/0.002	0.234/0.003	0.234/0.015	0.584/0.005
*t*/*P* 6 months treatment group value		0.471/0.013	0.422/0.014	0.742/0.003	0.234/0.023
*t*/*P* 12 months treatment group value		0.315/0.001	0.512/0.032	0.284/0.002	0.679/0.015

**Table 3:** Comparison of CI, FAB, WML, and FHI in the VaD, VCIND, and NCI patients ($\bar{x}$ ± *s*).

Group	Treatment time	CI	FAB	WML	FHI
VaD (*n* = 40)	Before treatment	5.92 ± 0.89	11.41 ± 2.34	14.15 ± 3.23	3.43 ± 0.63
	After 6 months of treatment	5.58 ± 0.81	11.57 ± 2.53	9.54 ± 2.35	3.75 ± 0.58
	After 12 months of treatment	5.14 ± 0.69	11.64 ± 2.77	3.63 ± 2.47	3.95 ± 0.29
VCIND (*n* = 40)	Before treatment	5.28 ± 0.84	11.45 ± 2.44	11.34 ± 3.16	3.55 ± 0.87
	After 6 months of treatment	5.16 ± 0.81	11.53 ± 2.34	7.68 ± 2.33	3.84 ± 0.64
	After 12 months of treatment	5.09 ± 0.78	11.57 ± 2.70	3.55 ± 2.45	4.01 ± 0.46
NCI (*n* = 40)	Before treatment	5.05 ± 0.88	11.44 ± 2.44	3.10 ± 3.55	4.06 ± 0.35
	After 6 months of treatment	5.03 ± 0.67	11.56 ± 2.83	2.34 ± 2.27	4.07 ± 0.51
	After 12 months of treatment	5.02 ± 0.66	11.63 ± 2.54	2.67 ± 2.68	4.09 ± 0.32
*F*/*P* VaD group value		0.758/0.001	0.660/0.014	0.116/0.016	0.855/0.001
*F*/*P* VCIND group value		0.431/0.011	0.357/0.006	0.775/0.007	0.465/0.015
*F*/*P* NCI group value		0.297/0.024	0.486/0.001	0.248/0.001	0.784/0.020
*t*/*P* 6 months treatment group value		0.644/0.001	0.355/0.022	0.351/0.014	0.694/0.010
*t*/*P* 12 months treatment group value		0.586/0.014	0.622/0.001	0.896/0.001	0.579/0.001

**Table 4:** Correlation analysis between neuropsychological scale score and degree of white matter lesions.

Indicators	VaD	VCIND	NCI	*t*	*P*
CSE	0.43	0.24	−0.35	17.78	0.26
MAS	−0.35	0.37	−0.59	10.64	0.13
CDT	0.71	0.68	0.66	46.66	0.27
DST	−0.29	0.55	0.19	23.17	0.09
FAB	0.32	0.46	0.22	19.35	0.01
VFT	−0.67	−0.74	−0.45	12.55	0.05
CWT	−0.54	−0.62	−0.84	10.28	0.25
RT	−0.39	−0.56	−0.75	11.45	0.06
TE	−0.21	−0.63	−0.53	14.46	0.13
T1W1	0.38	−0.30	0.74	16.85	0.04
T2W2	0.46	−0.28	0.11	13.57	0.06
DWI	0.57	−0.76	−0.54	36.68	0.03
FVO	−0.11	0.36	−0.64	44.58	0.12
TMT	−0.57	0.56	−0.78	15.43	0.01
CI	−0.33	0.76	0.63	14.66	0.31
WML	0.79	0.44	0.37	17.28	0.12
TVW	0.43	−0.12	0.45	15.69	0.09
FHI	0.29	−0.49	0.52	18.73	0.02

## Data Availability

Data sharing not applicable to this article as no datasets were generated or analyzed during the current study.
